# Comparison of Initial Visual Outcomes Following Keratorefractive Lenticule Extraction (KLEx) Using VisuMax 500 Versus VisuMax 800

**DOI:** 10.7759/cureus.89702

**Published:** 2025-08-09

**Authors:** Jonathan C Davis, Mina M Sitto, Eduardo Herrera, Bryce L Palmer, Phillip Hoopes, Majid Moshirfar

**Affiliations:** 1 Ophthalmology, University of South Carolina School of Medicine, Columbia, USA; 2 Ophthalmology, Hoopes Vision, Hoopes Vision Research Center, Draper, USA; 3 Ophthalmology, Wayne State University School of Medicine, Detroit, USA; 4 Ophthalmology, Albany Medical College, New York, USA; 5 Ophthalmology, Texas Tech University Health Sciences Center School of Medicine, Lubbock, USA; 6 Ophthalmology, Hoopes Vision, Draper, USA; 7 Ophthalmology, Cornea and Refractive Surgery, John A. Moran Eye Center, University of Utah School of Medicine, Salt Lake City, USA; 8 Eye Banking and Corneal Transplantation, Utah Lions Eye Bank, Murray, USA; 9 Ophthalmology, Hoopes Vision Research Center, Draper, USA

**Keywords:** astigmatism correction, keratorefractive lenticule extraction, lasik, nomogram, relex, second-generation klex, smile, smile pro, surgical innovation, vector analysis

## Abstract

Purpose

This study aims to compare the initial three-month outcomes of a single-center experience with small incision lenticule extraction (SMILE) for correction of myopia and myopic astigmatism using the VisuMax 500 (Carl Zeiss Meditec, Jena, Germany) versus the VisuMax 800 (SMILE Pro®; Carl Zeiss Meditec, Jena, Germany). This experience is compared to the US Food and Drug Administration approval studies and published literature.

Patients and methods

The initial 45 eyes (23 patients) that underwent SMILE with the VisuMax 500 in 2018 were compared with the initial 42 eyes (21 patients) that underwent SMILE Pro® with the VisuMax 800 in 2024. These patients represent the first to be treated with SMILE or SMILE Pro® at a single institution in Draper, Utah. Data from postoperative visits up to three months of follow-up were collected and analyzed. Generalized estimating equations (GEE) were used in our analysis to account for inter-eye bias.

Results

By three months, six eyes (13.3%) reported uncorrected distance visual acuity (UDVA) of 20/16 or better in the VisuMax 500 group compared to nine eyes (23.1%) in the VisuMax 800 group. All 87 eyes had a UDVA of 20/40 or better at three months. There was no statistically significant difference in the mean postoperative UDVA (logMAR) between platforms (0.02 ± 0.07 vs 0.04 ± 0.15; *P *= 0.315). Safety and efficacy indices were also comparable between platforms (*P *= 0.406 and 0.239, respectively). VisuMax 800 demonstrated less undercorrection of astigmatism compared to VisuMax 500; however, this was not statistically significant (*P *= 0.174).

Conclusion

Both platforms remain safe and effective for the treatment of myopia with and without astigmatism. Despite the improved ergonomic technology of the VisuMax 800, its three-month visual outcomes are comparable to those of the VisuMax 500.

## Introduction

Keratorefractive lenticule extraction (KLEx) was approved by the US Food and Drug Administration (FDA) in 2016 for the treatment of simple myopia using the VisuMax 500 Hz femtosecond laser system (Carl Zeiss Meditec, Jena, Germany) [1]. Compared with traditional laser in situ keratomileusis (LASIK) and photorefractive keratectomy (PRK), the first-generation KLEx can be used for the treatment of myopia and myopic astigmatism. Small incision lenticule extraction (SMILE) is a first-generation KLEx procedure that has been in use since 2008, with at least six million procedures performed globally [2,3]. In 2018, astigmatic software was developed for the treatment of cylinders up to -3.00 diopters (D), which allowed the VisuMax 500 to be approved for the treatment of myopic astigmatism [4].

Continued advancements led to the development of the second-generation SMILE using the VisuMax 800 femtosecond laser (Carl Zeiss Meditec, Jena, Germany), termed SMILE Pro®, which received FDA approval in 2024 [5]. While still containing the same laser elements as the VisuMax 500, the VisuMax 800 introduces several improvements over its predecessor, including an eye-tracking system, cyclotorsion compensation, and a reduction in total higher order aberrations (HOAs) [6]. However, as a recently approved platform, the VisuMax 800 has only been evaluated in a limited number of studies, and few compare its outcomes to those of other refractive procedures. Among the early studies available, visual outcomes show comparable results to those of the first-generation KLEx, despite the added features [6]. This study aims to directly compare the initial experience of 87 eyes undergoing treatment for myopia and myopic astigmatism with the first-generation VisuMax 500 versus the second-generation VisuMax 800, and to evaluate the visual outcomes with existing literature and the FDA clinical trials data.

## Materials and methods

A non-randomized, retrospective study was conducted on the first cohorts of patients who underwent SMILE and SMILE Pro® procedures performed by a single surgeon at a refractive surgery center in Draper, Utah. We included the initial 45 eyes (23 patients) treated with the VisuMax 500 between October 2018 and June 2019 and the initial 42 eyes (21 patients) treated with the VisuMax 800 between September 2024 and February 2025. Despite the retrospective nature of this study, it was approved by the Hoopes Vision Ethics Committee and the Biomedical Research Alliance of New York Institutional Review Board (#A20-12-547-823). The study adhered to the tenets of the Declaration of Helsinki. All participating patients provided written informed consent.

Inclusion criteria were as follows: the initial cohort of patients treated with SMILE or SMILE Pro® with at least a three-month postoperative follow-up, age ≥ 18 years, corrected distance visual acuity (CDVA) of 20/30 or better, and myopia (-8.50 to -2.25 D) with or without astigmatism (-2.50 D to 0.00 D). Patients were excluded if they had clinically significant dry eyes, ocular disease (retinal detachment, macular degeneration, keratoconus, glaucoma, or cataract formation), infectious or autoimmune disease (such as herpes simplex infection or systemic lupus erythematosus), hyperopic astigmatism, hyperopia, or if they had undergone previous corneal refractive surgery.

All patients underwent a comprehensive preoperative ophthalmic examination, including uncorrected distance visual acuity (UDVA), CDVA, intraocular pressure measurement, slit lamp biomicroscopy, dilated fundus examination, manifest refraction spherical equivalent (SEQ), and keratometry. Corneal topography and pachymetry were assessed using the Pentacam HR (Oculus Optikgeräte GmbH, Wetzlar, Germany). Postoperative evaluations were conducted at one day, one week, one month, and three months. Visual acuity was measured at each visit, while refractive outcomes were assessed at the one-month and three-month follow-ups.

Surgical procedure

For the SMILE platform, we used an established nomogram that was initially developed using Datagraph-med® version 5.80 (Datagraph, Chapel Hill, NC) for the treatment of simple myopia and astigmatism of ≤0.50 D prior to myopic astigmatic approval in 2018. This nomogram increased the sphere by 7.5% with no adjustment to the cylinder. In 2024, for SMILE Pro® using the VisuMax 800, the sphere was adjusted by 7.5%, while the cylinder was adjusted by 10%.

In the SMILE group, the VisuMax 500 kHz femtosecond laser system was used with pulse durations ranging from 220 to 580 fs and the following laser parameters: 3.93 mm superior corneal incision, small cone size, 7.5 mm cap diameter, 120 µm cap thickness, 6.0-6.5 mm lenticule diameter, superior incision position, 90° side-cut angle, 50° incision angle, laser-bed energy of 145 nJ, and spot separations of 3 µm (lenticule), 2.5 µm (side cut), 3 µm (cap), and 2 µm (incision side cut).

The SMILE Pro® group was treated with the VisuMax 800 kHz femtosecond laser system, utilizing the same parameters as the VisuMax 500 group with the exception of the following adjustments: 3.93 mm superior corneal incision, 60° incision angle, spot separation of 4 µm (lenticule), 2 µm (side cut), and 4 µm (cap).

Postoperative management consisted of one drop of moxifloxacin 0.5% ophthalmic solution in both eyes following the procedure. Patients continued moxifloxacin eye drops four times per day for one week and prednisolone acetate 1% ophthalmic suspension drops four times per day for one week, then twice a day for one week, and then once a day for two weeks postoperatively. Preservative-free artificial tears were recommended as needed.

Statistical analyses

Statistical analyses were performed using Python (v3.12.7; Python Software Foundation, Fredericksburg, VA) with NumPy (v1.26.4), SciPy (v1.15.3), Pandas (v2.2.3), and Statsmodels (v0.14.2) libraries. Given that randomly selecting one eye per patient would reduce our sample size, we chose to include both eyes from each patient in the analysis. To utilize both eyes, the general estimating equation (GEE) was used to adjust for potential inter-eye correlation and account for within-subject bias. The Shapiro-Wilk test was used to assess the normality of data distribution. An a priori power analysis using R software (v4.3.2; R Foundation for Statistical Computing, Vienna, Austria) with an alpha of 0.05, power of 0.8, effect size of 0.13 logMAR UDVA, standard deviation of 0.09 logMAR [7], intra-cluster correlation of 0.9 [8], and a mean cluster size of 1.977 indicated a minimum sample size of 15 patients (30 eyes) for SMILE and 15 patients for SMILE Pro® (30 eyes). A *P*-value of less than 0.05 was considered statistically significant.

Vector analysis

Astigmatic vector analysis was performed in accordance with the Alpins method [9-12]. Manifest refraction values were converted to the corneal plane using a back-vertex distance of 12 mm. The intended astigmatic change was defined as the target-induced astigmatism (TIA), and the actual astigmatic correction was defined as the surgically induced astigmatism (SIA), with emmetropia as the refractive goal. The difference between TIA and SIA is the difference vector (DV), and the magnitude of error (ME) is the arithmetic difference between TIA and SIA. The angle of error represents the angular difference between TIA and SIA, and the corrective index (CI) is the ratio of SIA to TIA and has an ideal value of 1. The mean of all CI values was calculated using the geometric mean.

Selection of published literature for SMILE versus SMILE Pro®

To facilitate comparison with existing studies, a literature search was conducted to identify studies that compare the visual outcomes of VisuMax 500 and VisuMax 800 for the treatment of myopia and myopic astigmatism (Table 1). The following search phrases were used: (“VisuMax 800” OR “Smile Pro” OR “second-generation KLEx”) AND (“keratorefractive lenticule extraction” OR “KLEx” OR “small incision lenticule extraction” OR “SMILE” OR “VisuMax 500”) AND (“myopia” OR “astigmatism”). This yielded 41 total results, including 11 studies from PubMed and 30 from SCOPUS. Of these, 37 were excluded because they were duplicates, not relevant to the study objective, or did not compare the VisuMax 800 and VisuMax 500 platforms. The four remaining comparative studies were included in our analysis [6,13-15]. In addition, we included the VisuMax 500 FDA Premarket Approval (PMA) data and the VisuMax 800 post-market clinical trials, given the objective results and sufficiently large sample sizes [5,16]. The following visual parameters were evaluated: safety, efficacy, predictability (SEQ accuracy), and astigmatic correction.

**Table 1:** Summary of selected literature reporting visual outcomes of VisuMax 500 and VisuMax 800 platforms Sph: Sphere; cyl: Cylinder; SEQ: Spherical equivalent; D: Diopters; UDVA: Uncorrected distance visual acuity; CDVA: Corrected distance visual acuity; VM500: VisuMax 500; VM800: VisuMax 800. Values are written as mean ± standard deviation (range, if provided). - indicates no available data. * indicates the postoperative sample size that differed compared to the preoperative sample size.

				Preoperative			Postoperative													
Study (year)	Country	Follow-up (months)	N	Sph (D)	Cyl (D)	SEQ (D)	Sph (D)	Cyl (D)	SEQ (D)	% Cyl ≤ 0.50 D	SEQ within ± 0.50 D	% UDVA ≥ 20/20	% UDVA ≥ 20/40	% Loss of 2 lines CDVA	% Loss of 1 line CDVA	% No change CDVA	% Gain of 1 line CDVA	% Gain of 2 lines CDVA	Safety index	Efficacy index
VisuMax 500 vs VisuMax 800 studies																				
Current study (2025)																				
VM500	US	3	45	-5.06 ± 1.73 (-8.50 to -2.25)	-0.74 ± 0.68 (-2.50 to 0.00)	-5.43 ± 1.78 (-8.63 to -2.25)	0.26 ± 0.35 (-0.25 to 1.50)	-0.39 ± 0.39 (-2.0 to 0.0)	0.06 ± 0.30 (-0.50 to 0.75)	88.9%	95.6%	87.0%	100.0%	0.0%	2.2%	75.6%	22.2%	0.0%	1.00 ± 0.03	0.99 ± 0.05
VM800	US	3	42	-5.04 ± 1.50 (-7.50 to -2.50)	-0.76 ± 0.69 (-2.25 to 0.00)	-5.42 ± 1.58 (-7.88 to -2.88)	-0.04 ± 0.43 (-1.25 to 0.75)	-0.35 ± 0.39 (-1.25 to 0.00)	-0.21 ± 0.45 (-1.75 to 0.50)	81.0%	90.5%	77.0%	100.0%	0.0%	2.4%	69.0%	26.2%	2.4%	1.00 ± 0.04	0.97 ± 0.08
Yoo et al. (2024) [6]																				
VM500	South Korea	6	100	-3.61 ± 1.15 (-6.87 to -1.25)	-0.78 ± 0.63 (-2.25 to 0.0)	-	-	-	-	94.0%	97.0%	93.0%	100.0%	0.0%	0.0%	33.0%	65.0%	2.0%	-	-
VM800	South Korea	6	50	-3.63 ± 1.42 (-6.75 to -1.12)	-0.78 ± 0.53 (-2.25 to -0.25)	-	-	-	-	94.0%	98.0%	94.0%	100.0%	0.0%	0.0%	48.0%	46.0%	6.0%	-	-
Lee et al. (2024) [13]																				
VM500	Taiwan	3	31	-4.9 ± 2.51	-1.77 ± 0.97	-5.79 ± 2.72	-	-0.80 ± 0.39	-0.61 ± 0.51	32.3%	51.6%	69.0%	100.0%	0.0%	24.0%	76.0%	0.0%	0.0%	0.95 ± 0.09	-
VM800	Taiwan	3	35	-5.64 ± 2.25	-1.85 ± 1.12	-6.57 ± 2.49	-	-0.50 ± 0.41	0.66 ± 0.51	62.9%	51.4%	80.0%	100.0%	0.0%	3.0%	94.0%	3.0%	0.0%	1.00 ± 0.06	-
Ganesh et al. (2025) [14]																				
VM500	India	3	30	-3.82 ± 1.95	-0.74 ± 0.53	-3.82 ± 1.95	-	-	-	100.0%	97.0%	93.0%	100.0%	0.0%	0.0%	68.0%	32.0%	0.0%	-	-
VM800	India	3	30	-3.85 ± 1.86	-0.80 ± 0.63	-3.85 ± 1.86	-	-	-	97.0%	100.0%	97.0%	100.0%	0.0%	0.0%	77.0%	23.0%	0.0%	-	-
Varman et al. (2024) [15]																				
VM500	India	3	115	-3.56 ± 2.11 (-9.25 to -0.50)	-2.54 ± 1.06 (-5.00 to -1.50)	-4.82 ± 2.16 (-10.25 to -1.25)	-0.18 ± 0.21 (-0.50 to 0.25)	0.27 ± 0.31 (0.00 to 1.00)	-0.32 ± 0.20 (-0.88 to 0.37)	82.0%	71.0%	94.0%	100.0%	0.0%	4.0%	65.0%	30.0%	0.0%	-	-
VM800	India	3	105	-3.55 ± 2.26 (-10.50 to 0.50)	-2.49 ± 0.79 (-4.75 to -1.50)	-4.80 ± 2.24 (-12.00 to -0.75)	-0.15 ± 0.22 (-0.75 to 0.50)	0.14 ± 0.29 (0.00 to 0.75)	-0.27 ± 0.22 (-1.00 to 0.38)	92.0%	96.0%	98.0%	100.0%	0.0%	3.0%	92.0%	5.0%	0.0%	-	-
VisuMax 500 studies																				
FDA Premarket Approval (2018) [4]	United States	12	357*	-4.82 ± 2.39 (-10.0 to -1.00)	-1.34 ± 0.80 (-3.00 to 0.00)	-	-	-	-	91.6%	94.8%	89.4%	98.9%	0.0%	2.3%	73.6%	22.3%	1.4%	-	-
VisuMax 800 studies																				
Sekundo et al. (2025) [5]																				
VM800	Germany	6	452*	-4.06 ± 1.85 (-9.00 to 0.00)	0.87 ± 0.71 (0.00 to 3.75)	-4.49 ± 1.87 (-10.00 to -0.25)	-	-	-	90.8%	95.1%	93.3%	100.0%	0.0%	9.8%	57.8%	30.9%	1.6%	1.07 ± 0.14	0.99 ± 0.18
Reinstein et al. (2025) [16]																				
VM800	England	3	118*	-4.16 ± 1.89 (-8.92 to -1.02)	-0.98 ± 0.78 (-4.00 to 0.00)	-4.65 ± 2.01 (-11.17 to -2.17)	-	-	-	85.0%	86.0%	91.0%	100.0%	0.0%	8.0%	76.0%	15.0%	0.0%	-	-

## Results

Patient demographics

Preoperative characteristics of both platforms are shown in Table 2. The mean age was 32.4 ± 7.8 years (range: 18 to 57) for the VisuMax 500 group and 33.8 ± 6.3 years (range: 22 to 46) for the VisuMax 800 group (*P* = 0.113). The VisuMax 500 group included 12 (52.2%) males and 11 (47.8%) females, and the VisuMax 800 group included 11 (52.4%) males and 10 (47.6%) females (*P* = 0.989). There were no statistically significant differences in UDVA, SEQ, sphere, or cylinder between groups preoperatively (Table 2).

**Table 2:** Preoperative characteristics UDVA: Uncorrected distance visual acuity; SEQ: Spherical equivalent; D: Diopters. Values are expressed as mean ± standard deviation (range).

Parameters	VisuMax 500	VisuMax 800	P
N (eyes)	45	42	
Sex, Male/Female (%)	52.2%/47.8%	52.4%/47.6%	0.989
Age (years)	32.4 ± 7.8 (18 to 57)	33.8 ± 6.3 (22 to 46)	0.113
UDVA (logMAR)	1.38 ± 0.32 (0.40 to 1.80)	1.41 ± 0.26 (0.70 to 1.80)	0.657
SEQ (D)	-5.43 ± 1.78 (-8.63 to -2.25)	-5.42 ± 1.58 (-7.88 to -2.88)	0.982
Sphere (D)	-5.06 ± 1.73 (-8.50 to -2.25)	-5.04 ±1.50 (-7.50 to -2.50)	0.955
Cylinder (D)	-0.74 ± 0.68 (-2.50 to 0.00)	-0.76 ± 0.69 (-2.25 to 0.00)	0.875

Efficacy

**Figure 1:**
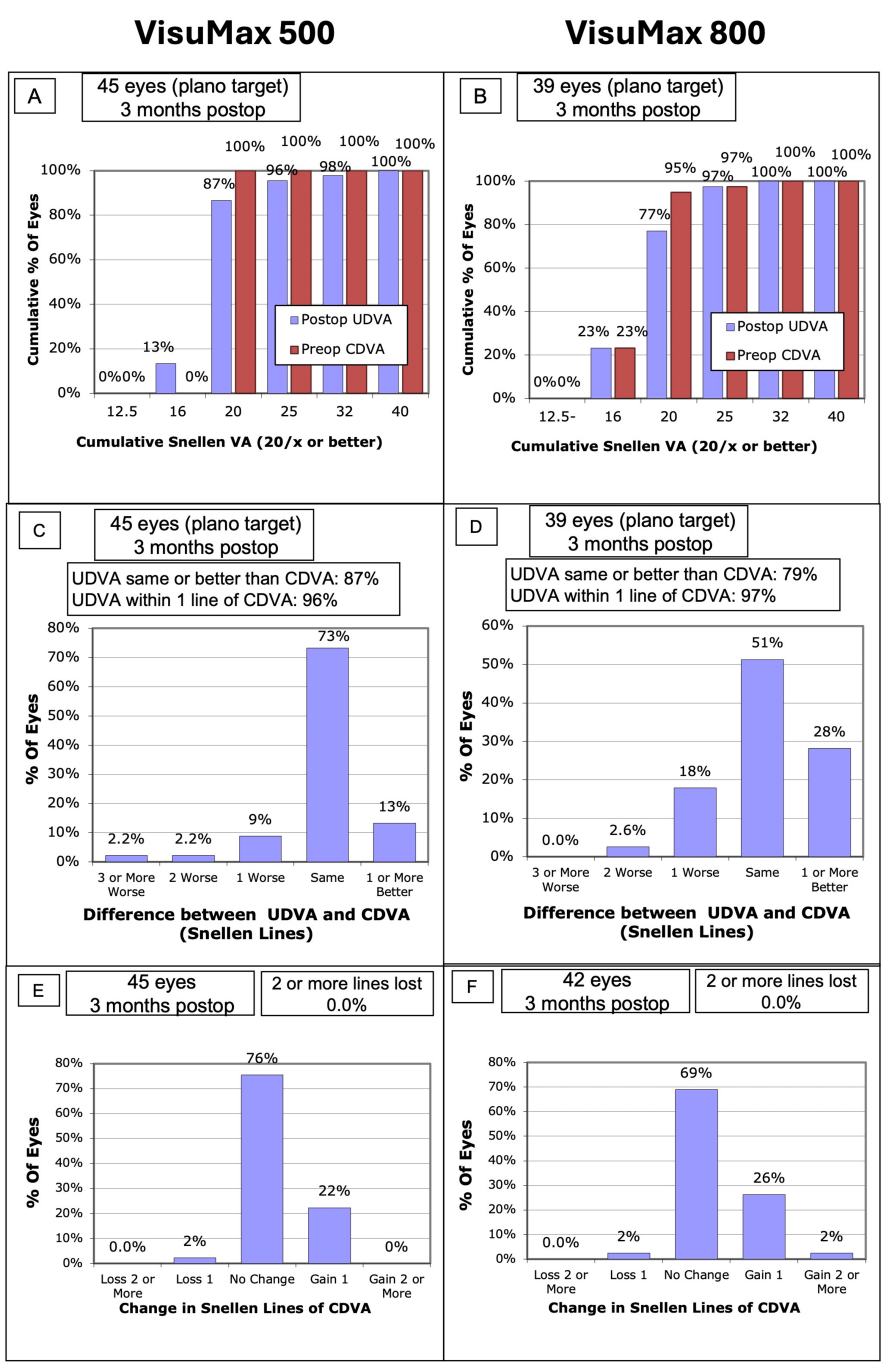
Comparison of safety and efficacy between the VisuMax 500 (Panels A, C, and E) and VisuMax 800 (Panels B, D, and F) at three months postoperatively, based on the nine standard graphs Panels A and B show cumulative Snellen visual acuity. Panels C and D compare postoperative uncorrected distance visual acuity (UDVA) with preoperative corrected distance visual acuity (CDVA). Panels E and F show the change in CDVA from preoperative to three months postoperative.

All eyes were included in the visual acuity comparative analysis except those with a target other than plano. By three months, six eyes (13.3%) in the VisuMax 500 group reported 20/16 or better UDVA compared to nine eyes (23.1%) in the VisuMax 800 group (Figure 1, Panels A and B). Both groups had 100% of eyes with UDVA of 20/40 or better. There was no statistically significant difference in the mean postoperative UDVA (logMAR) between platforms (0.02 ± 0.07 vs 0.04 ± 0.15; *P* = 0.315). In the VisuMax 800 group, 11 eyes (28.2%) achieved a postoperative UDVA of one line or better than baseline CDVA, whereas only six eyes (13.3%) in the VisuMax 500 group achieved this outcome (Figure 1, Panels C and D). Efficacy indices for VisuMax 500 and VisuMax 800 were 0.99 ± 0.05 and 0.97 ± 0.08, respectively (*P* = 0.239), with no significant difference (Table 1).

Safety

In terms of safety, 12 eyes (28.6%) in the VisuMax 800 group gained one or two lines of CDVA, including one eye (2.4%) that gained two lines of CDVA (Figure 1, Panel F). The VisuMax 500 group had 10 eyes (22.2%) that gained one line of CDVA; however, no eyes gained two lines of CDVA (Figure 1, Panel E). Safety indices for both VisuMax 500 and VisuMax 800 were 1.00 (*P* = 0.406) (Table 1).

Accuracy and stability

**Figure 2:**
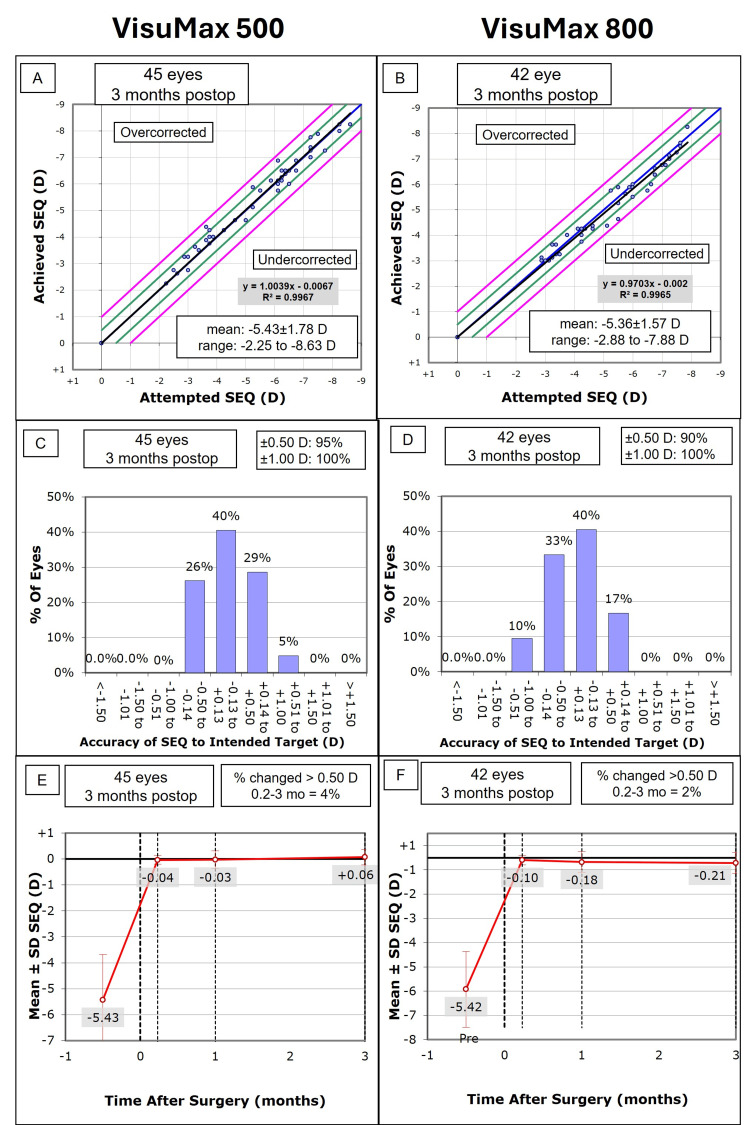
Comparison of spherical equivalent (SEQ) accuracy and stability between the VisuMax 500 (Panels A, C, and E) and VisuMax 800 (Panels B, D, and F) at three months postoperatively, based on the nine standard graphs Panels A and B show attempted versus achieved SEQ. Panels C and D show the accuracy of SEQ to the intended target. Panels E and F demonstrate the stability of SEQ from preoperative to three months postoperative.

All eyes in both groups were within ±1.00 D of the SEQ target at three months postoperatively (Figure 2, Panels C and D). However, 43 eyes (95.6%) in the VisuMax 500 group were within ±0.50 D of target SEQ compared to 38 eyes (90.5%) in the VisuMax 800 group. At three months, the mean SEQ in the VisuMax 800 group was significantly more myopic than the VisuMax 500 group (-0.21 ± 0.45 D vs 0.06 ± 0.30 D; *P* < 0.001) (Table 1). Similarly, the VisuMax 800 had a mean sphere of -0.04 ± 0.43 D, which was significantly more myopic than the VisuMax 500 group (0.26 ± 0.35 D; *P *< 0.001). In terms of SEQ stability from one to three months postoperatively, two eyes (4.4%) in the VisuMax 500 group and one eye (2.4%) in the VisuMax 800 group experienced a change of more than 0.50 D (Figure 2, Panels E and F).

Astigmatism analysis

**Figure 3:**
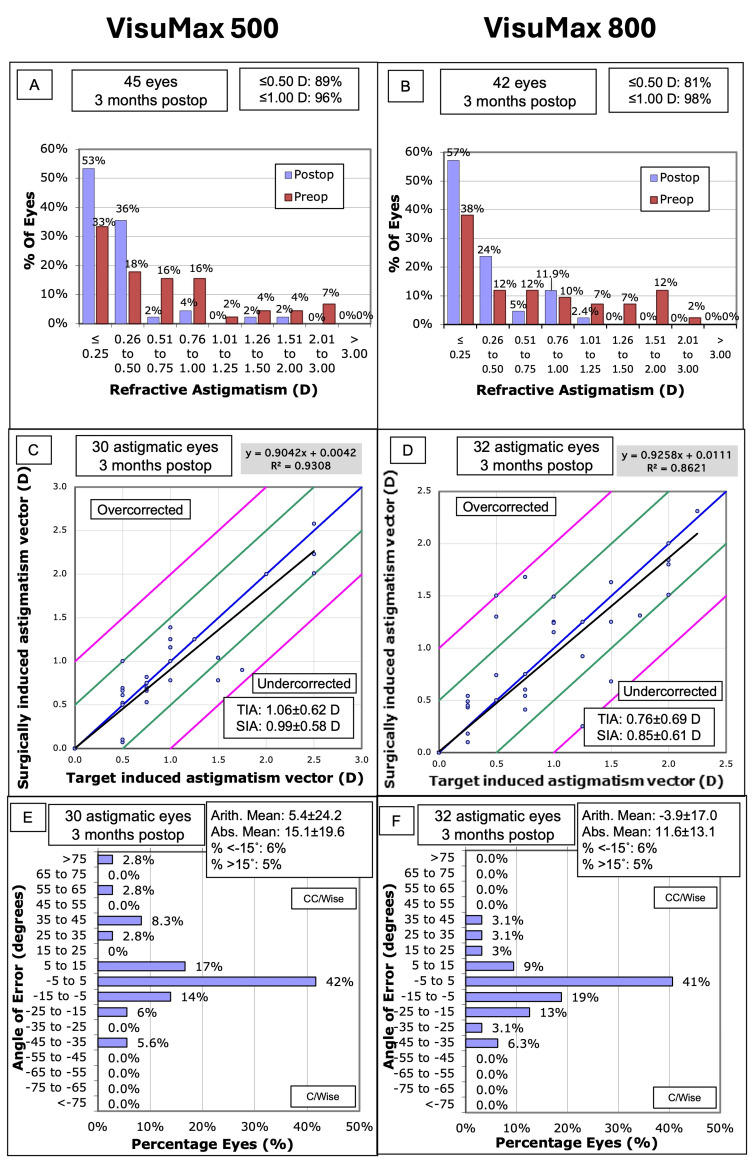
Standard graphs for astigmatism correction at three months postoperatively between the VisuMax 500 (Panels A, C, and E) and VisuMax 800 (Panels B, D, and F) Panels A and B show the distribution of refractive astigmatism from baseline to three months. Panels C and D show the correlation between target-induced (TIA) and surgically induced astigmatism (SIA). Panels E and F show the distribution of the angle of error in refractive astigmatism.

In the VisuMax 500 group, 40 eyes (88.9%) had a cylinder of ≤0.50 D at three months (Figure 3, Panel A). In comparison, 34 eyes (81.0%) in the VisuMax 800 group achieved a cylinder of ≤0.50 D (Figure 3, Panel B). Postoperative cylinder was comparable between the VisuMax 500 and VisuMax 800 platforms (-0.39 ± 0.39 D vs -0.35 ± 0.39 D, respectively; *P* = 0.599) (Table 1).

**Figure 4:**
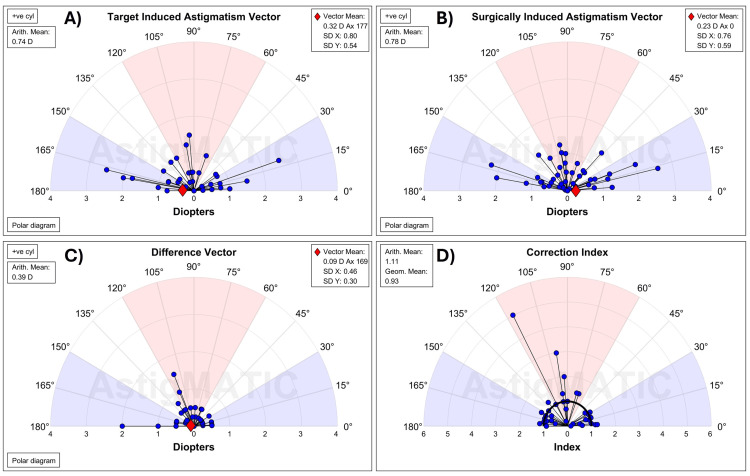
Vector analysis for the VisuMax 500

**Figure 5:**
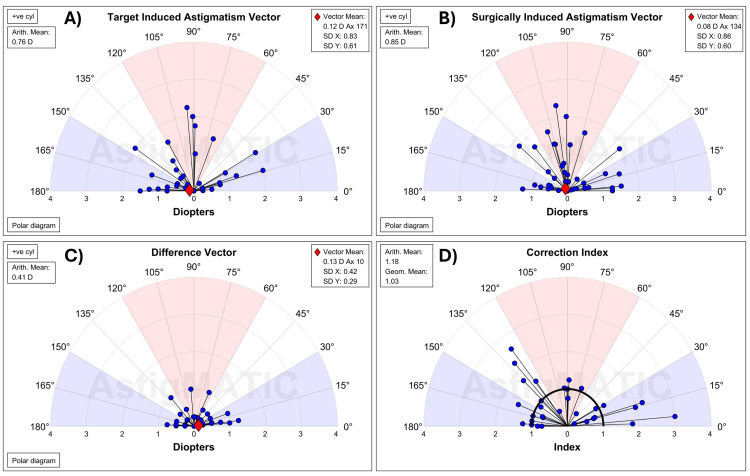
Vector analysis for the VisuMax 800

Following vector analysis, the mean TIA of the VisuMax 500 and VisuMax 800 groups was 0.74 D and 0.76 D, respectively (Figures 4, 5). At three months postoperatively, the SIA was 0.78 D in the VisuMax 500 group and 0.85 D in the VisuMax 800 group. The correlation between TIA and SIA showed a coefficient of determination (*R*²) of 0.93 for VisuMax 500 and 0.86 for VisuMax 800 (Figure 3, Panels C and D). The geometric mean of CI at three months for VisuMax 500 eyes was 0.93 (Figure 4), corresponding to a slight undercorrection. Conversely, the geometric mean of the CI for VisuMax 800 eyes was 1.03 (Figure 5), indicating slight overcorrection, though this was not significantly different (*P* = 0.174). The angle of error astigmatism was more tightly distributed in the VisuMax 800 eyes, with values ranging from -35° to 45° (Figure 3, Panel F). Conversely, the VisuMax 500 eyes had a wider distribution for angle of error, with a greater proportion of eyes near the extremes (Figure 3, Panel E). This was evidenced by the absolute mean angle of error, which was greater in the VisuMax 500 (15.1 ± 19.6°) compared to the VisuMax 800 (11.6 ± 13.1°) (Figure 3, Panels E and F).

## Discussion

The present study is among the earliest US studies to report visual and refractive outcomes from the initial eyes treated with the VisuMax 500 and VisuMax 800 platforms. Both groups demonstrated excellent and comparable clinical outcomes, which is consistent with the findings reported by Yoo et al. [6]. However, our study showed significantly more hyperopic sphere in the VisuMax 500 group compared to the VisuMax 800 group (*P* < 0.001). Although the VisuMax 500 showed a greater magnitude of cylinder postoperatively, this was not statistically significant (*P* = 0.599). Together, the spherical and cylindrical values in the VisuMax 500 group resulted in a lower mean SEQ closer to emmetropia than the VisuMax 800 group (*P* < 0.001).

In the VisuMax 500 group, our findings are consistent with those of the FDA PMA study, which reported that 294 of 353 eyes (83.3%) achieved a postoperative UDVA of 20/20 or better at three months [4]. Similarly, nearly all eyes in both our study and the FDA clinical trial showed no change or gain in CDVA lines (Table 1). Conversely, the VisuMax 800 results differ from those of the FDA post-market clinical trial and the original study by Reinstein et al. [5,16]. The lower proportion of eyes achieving a postoperative UDVA of 20/20 in our study may be attributed to the smaller sample size, where a few more eyes with a three-month UDVA of 20/25 disproportionately impacted the overall percentage. Additionally, although we excluded eyes that required enhancements for the VisuMax 500, insufficient time has passed to determine whether patients in the VisuMax 800 group will also require enhancements. Nonetheless, all 87 eyes in our study achieved a UDVA of 20/40 or better at three months (Table 1).

Both the VisuMax 500 and VisuMax 800 groups had one eye (2.2% to 2.4%) each with a loss of one CDVA line, with no eyes losing two or more lines (Figure 1, Panels E and F). Similarly, all other published studies reported no eyes losing two or more lines for CDVA (Table 1). In addition, the safety and efficacy indices for both platforms were comparable (*P* = 0.406 and 0.239, respectively), supporting KLEx as a safe and effective procedure for treating myopia with or without astigmatism, regardless of whether SMILE or SMILE Pro® is used.

A notable trend observed in our study was the tendency for the VisuMax 500 to undercorrect astigmatism, as evidenced by a geometric mean for CI below 1.00 (Figure 5, Panel A). Conversely, the VisuMax 800 had a geometric mean for CI slightly above 1.00 (Figure 5, Panel B), indicating a slight overcorrection, although this was not significantly different (*P* = 0.174). Our findings are consistent with those reported by Zhang et al., who suggested that nomograms should be adjusted to account for the undercorrection of astigmatism in SMILE [17].

Nomograms were developed to achieve more optimal results by compensating for the subtle differences between surgeons, lasers, institutions, and patient characteristics [18]. Liang et al. validated that the implementation of a nomogram improves the predictability of SMILE for target refractive values [19]. The differences in the characteristics of the nomograms used for VisuMax 500 and VisuMax 800 may contribute to the observed variations in clinical outcomes. Notably, the VisuMax 800 nomogram has been extensively refined, with six additional years of accumulated patient outcome data, including data from eyes treated with the VisuMax 500. Therefore, this may explain the observed undercorrection of astigmatism in the VisuMax 500 eyes treated in 2018 when limited data were available, compared to more accurate corrections seen in the VisuMax 800 eyes treated in 2024 to 2025.

Although the laser components and optical systems are identical, the VisuMax 800 has several mechanical advantages that may contribute to its overall improved outcomes. One advantage is the reduced procedure time compared to the VisuMax 500, with multiple studies reporting that the VisuMax 800 is significantly faster in terms of lenticule creation and overall treatment durations [6,14]. This increased speed has been especially beneficial during the critical steps of the procedure, such as lenticule creation, which may benefit more anxious patients who would otherwise be contraindicated for KLEx using the VisuMax 500 [14]. Additionally, given that the risk of suction loss is directly related to the length of the procedure, the shorter overall treatment time with the VisuMax 800 may reduce this adverse event. Though we did not assess patient-reported outcomes, previous studies have shown that patients and surgeons alike prefer the VisuMax 800, likely due to its increased speed and improved user experience [14].

One limitation that should be acknowledged is the single-center, retrospective design of the study, which may introduce selection or information bias. The addition of OcuLign® in VisuMax 800 did not show the expected improvement in astigmatic correction, which may be attributed to the surgeon’s years of experience at our institution. The initial learning curve of SMILE is particularly challenging as it requires manual control of cyclotorsion [20]. Additionally, the manual marking technique used with the VisuMax 500 at our institution has helped mitigate the difficulty of manual cyclotorsion. Another factor that may have contributed to the comparable outcomes between the two platforms is the refinement of our nomogram. These may contribute to the diminished benefit that OcuLign® provides in the newer model at our single institution. For surgeons with limited experience with SMILE or for correction of severe myopic astigmatism, the addition of OcuLign® may lead to a significantly different outcome between VisuMax 500- and VisuMax 800-treated eyes, which was not observed in this study. Another limitation of our study is that we did not compare the induction of HOAs following the SMILE procedure. Yoo et al. reported that VisuMax 800 induced fewer total HOAs [6]. This comparison should be explored further as additional outcome data becomes available.

The sample size of our study is important to consider when interpreting the results. Although we included only 87 eyes for comparison (45 for VisuMax 500 and 42 for VisuMax 800), a post hoc power analysis confirmed an overall statistical power of 0.937. This reflects a sufficiently robust sample size for detecting a statistically significant difference between platforms. However, based on our initial three-month experience, both SMILE and SMILE Pro® demonstrate similar safety, efficacy, and predictability for the treatment of myopia with or without astigmatism.

## Conclusions

Despite the ergonomic advancements of the recently introduced VisuMax 800, our findings demonstrate that the visual outcomes remain comparable to those of its predecessor, the VisuMax 500. Future studies with larger cohorts and extended follow-up durations are required to elucidate subtle differences in visual outcomes. We anticipate that as the VisuMax 800 becomes more widely adopted, more comprehensive comparative data will help determine whether these added features provide a clinically meaningful advantage.
